# Supplementary material for: Experiences of persons with Multiple Sclerosis with lifestyle adjustment–A qualitative interview study
Source: PLoS One. 2022 May 27;17(5):e0268988. doi: 10.1371/journal.pone.0268988 (PMC9140290; doi:10.1371/journal.pone.0268988)
Supplement: S1 Appendix — (DOCX) [file pone.0268988.s001.docx]

**S1 Appendix. Interview guide.**

| Guiding questions | Check: Was that mentioned? | Specific questions |
| --- | --- | --- |
| I. I would like you to tell me about your life with MS. The best way to begin is to start with how you were diagnosed with MS.  You can take your time, even for details, because everything that is important to you is interesting to me. | - Symptoms and indications before diagnosis - Medical examinations - Diagnosis |  |
| II Please tell me if you have informed people around you that you have MS.  If so, what was it like when you told them? | - Who has the pwMS told that he or she has MS? - When did the pwMS tell about MS? | - How did you tell those in your social environment? - When did you tell them? - Who did you tell? - Who were you worried about whether or not to tell about your illness? |
| III. Please tell me about your experience with DMTs. | Which DMTs were used?   - Glatiramer acetate - Inteferon-beta - Dimethyl fumarate - Teriflunomide - Alemtuzumab - Daclicumab - Fingolimod - Mitoxantrone - Natalizumab - Cladribine - Ocrelizumab   Handling and side effects | Can you tell me how it was for you when you had to decide on a DMT or against a DMT?  Question to pwMS who have taken DMTs:   - Have you had any problems with the DMT? - Can you tell me how you are taking/took this medicine and how you are coping/coped with it?   Question to pwMS who have not taken DMTs:   - How did you feel about not taking a DMT? - Did you have any problems with not taking a DMT? |
| IV. Apart from DMTs, there are other therapies that can be used. These include alternative therapies or measures that change lifestyle habits.  If you have tried anything, please tell me about your experiences with it. | Alternative therapies, e.g. homeopathy, acupuncture, treatments on biological basis  Lifestyle measures, e.g. nutrition, exercise and sports, relaxation and stress management | - What have you already tried out and how did you experience it? - How did you decide to do this? |
| V. What experiences have you had with rehabilitation methods? | Outpatient rehabilitation?  Inpatient rehabilitation? | If rehabilitation was done:   - Which impact did the rehabilitation have on you? - How did you decide to do rehabilitation?   If no rehabilitation was done:   - Was that a topic of discussion at any point? - If so: Can you tell me about how you decided against rehabilitation? |
| VI. Could you please tell me about your everyday life with MS. | - - Work life; disability and retirement   - Social life, stigmatisation   - Pregnancy, birth, children   - Adaptation and aids   - Mobility, Travel   - Nutrition, food supplements   - Exercise, sports   - Relaxation / stress management |  |
