# Supplementary material for: Experiences of persons with Multiple Sclerosis with lifestyle adjustment–A qualitative interview study
Source: PLoS One. 2022 May 27;17(5):e0268988. doi: 10.1371/journal.pone.0268988 (PMC9140290; doi:10.1371/journal.pone.0268988)
Supplement: S2 Appendix — (DOCX) [file pone.0268988.s002.docx]

**Consolidated criteria for reporting qualitative studies (COREQ): 32-item checklist**

Developed from:

Tong A, Sainsbury P, Craig J. Consolidated criteria for reporting qualitative research (COREQ): a 32-item checklist for interviews and focus groups. *International Journal for Quality in Health Care*. 2007. Volume 19, Number 6: pp. 349 – 357

**DOMAIN 1: Research team and reflexivity**

| **Items** | **Guide question/description** | **Reported on Page #** |
| --- | --- | --- |
| *Personal characteristics* | | |
| Interviewer/facilitator | Which author/s conducted the interview or focus group? | P. 7:  “All interviews were conducted by a single interviewer (A.S.) – a female health scientist with expertise in qualitative research methods, who had no relationship to the patients in our study.” |
| Occupation | What was their occupation at the time of the study? |  |
| Gender | Was the researcher male or female? |  |
| Experience and training | What experience or training did the researcher have? |  |
| *Relationship with participants* | | |
| Relationship established | Was a relationship established prior to study commencement? | P. 7:  “[…] who had no relationship to the patients in our study.” |

**DOMAIN 2: Study design**

| **Items** | **Guide question/description** | **Reported on Page #** |
| --- | --- | --- |
| *Theoretical framework* | What methodological orientation was stated to underpin the study? e.g. grounded theory, discourse analysis, ethnography, phenomenology, content analysis | P. 7:  “Data were analyzed inductively and deductively according to the six-step (reflexive) thematic analysis of Braun and Clarke [43-46].” |
| *Participant selection* | | |
| Sampling | How were participants selected? | P. 6:  “For this study, a maximum variation sampling strategy [40] was applied to gather heterogeneous experiences with different MS therapeutic approaches. Participants were recruited from MS support groups, clinics, and rehabilitation centers. The inclusion criteria were (a) age ≥ 18 years and (b) a diagnosis of RRMS. PwMS with PPMS, poor knowledge of the German language, and severe cognitive impairments based on clinical impression were excluded from the study. Notably, the transition of RRMS to SPMS often covers a period of uncertainty, which can lead to difficulties in reliable distinction of RRMS and SPMS [41]. Hence, patients who were initially considered RRMS were identified as rather SPMS during the interview process. However, they were kept and questions referred to their experiences when having RRMS.” |
| Sample size | How many participants were in the study? | P. 8:  “There were 50 pwMS who participated (Table 1).” |
| *Setting* | | |
| Setting of data collection | Where was the data collected? e.g. home, clinic, workplace | P. 7:  “To include pwMS from all over Germany, and those with impaired mobility, the interviews were carried out in different locations on interviewees’ choice like participants’ homes and workplaces, clinics, rehabilitation centers, or hotels.” |
| Description of sample | What are the important characteristics of the sample? e.g. demographic data, date | P. 8:  Table 1. Demographic and MS-related characteristics of participants. |
| *Data collection* | | |
| Interview guide | Were questions, prompts, guides provided by the authors? Was it pilot tested? | P. 6:  “Qualitative data were collected by means of problem-centered, audio and video recorded interviews according to Witzel et al. [43] including mainly open questions on patients’ experiences of coping with the diagnosis of MS, of how to live with MS in everyday life, and of different treatment approaches (S1 Appendix).”  P. 6:  “A pilot run with five interviewees was performed to check for the comprehensibility of the questions and an adequate duration of the interview guide.” |
| Audio/visual recording | Did the research use audio or visual recording to collect the data? | P. 6:  “[…] audio and video recorded interviews […]” |
| Duration | What was the duration of the inter views or focus group? | P. 7:  “Interviews ranged from 20 to 97 minutes (mean 45.6 minutes).” |
| Data saturation | Was data saturation discussed? | P. 22:  “Data collection focused on DMTs rather than lifestyle adjustments. In particular, it would not have been impossible to define a saturation endpoint because of the many individual lifestyle choices.” |

**DOMAIN 3: Analysis and findings**

| **Items** | **Guide question/description** | **Reported on Page #** |
| --- | --- | --- |
| *Data analysis* | | |
| Number of data coders | How many data coders coded the data? | P. 7:  “Data were analyzed inductively and deductively according to the six-step thematic analysis of Braun and Clarke [44] by two researchers (S.EW., A.S.) using the software program MAXQDA Analytics Pro 2018.” |
| Derivation of themes | Were themes identified in advance or derived from the data? | P. 7:  “Data were analyzed inductively and deductively […]” |
| Software | What software, if applicable, was used to manage the data? | P. 7:  “[…]using the software program MAXQDA Analytics Pro 2018.” |
| *Reporting* | | |
| Quotations presented | Were participant quotations presented to illustrate the themes/findings? Was each quotation identified? e.g. participant number | In the section “Results” and in the Appendix we have presented quotations for each theme and identified each quotation with a participant number. |
| Clarity of major themes | Were major themes clearly presented in the findings? | In the section “Results” we have shown Fig 1-3, which illustrate each major theme and sub-theme (minor theme). Furthermore, we described each theme within the text in the Results in more detail. |
| Clarity of minor themes | Were minor themes clearly presented in the findings? |  |
