# Supplementary material for: Experiences of persons with Multiple Sclerosis with lifestyle adjustment–A qualitative interview study
Source: PLoS One. 2022 May 27;17(5):e0268988. doi: 10.1371/journal.pone.0268988 (PMC9140290; doi:10.1371/journal.pone.0268988)
Supplement: S3 Appendix — (DOCX) [file pone.0268988.s003.docx]

**S2 Appendix.** Themes, sub-themes and the corresponding exemplary quotes from pwMS.

| Experiences with Nutrition and Supplements\Starting new habits\Influencing factors\Information and advice | I am also in a few forums, on Facebook, where everything is discussed, how something could help [...]. And I just pick up the things and try it and see how my body reacts to it. [pwMS 04] |
| --- | --- |
|  | I am very grateful to my former boss. He called me a short time after I was diagnosed and said, "Take vitamin D." And I asked "yes why?". And he never explained well, but I knew after 10 years under his leadership, if he says something, then it has proof. So I started with vitamin D. [pwMS 32] |
|  | Yes, I did a nutritional consultation in the practice of one of the nutrition docs. Then there they advised me to eat a lot of high-fat dairy products because you need calories. [pwMS 32] |
|  | What I additionally use in terms of medication, also from a medical point of view, [are] vitamins, vitamin B and vitamin D supplements [...] which are supposed to somewhat counteract the fatigue. [pwMS 25] |
|  | What I have done before and will do again now is interval fasting, because that is also being tested in this nutrition study at Charité. [pwMS 32] |
| Experiences with Nutrition and Supplements\Starting new habits\Influencing factors\Active disease management | [I] ate something [...] in cream sauce [...], afterwards I had numb hands, completely and utterly. [...] I've always had the feeling […] that symptoms are worse or less bad depending on what I eat. [pwMS 36] |
|  | My diet consists of low linoleic acid food, because these very linoleic acids can be blamed for destroying the myelin layer. [pwMS 50] |
|  | But I try to reduce it as much as possible, because pork is always highly criticized for inflammation. [pwMS 38] |
|  | Since February, I've been trying to avoid sugar so that the [...] sugar [...] doesn't feed inflammation. [pwMS 40] |
|  | I thought I have to do something to give myself the feeling that I was doing something. [...] just this feeling of sitting there and doing nothing, and to wait for everything to get worse, that was something I [...] couldn't stand very well. [pwMS 36] |
| Experiences with Nutrition and Supplements\Starting new habits\Influencing factors\Desire for general health benefits | When I eat, then actually mostly only vegetables, always a protein carrier in addition. I try to avoid carbohydrates. Of course, with me the context is also weight loss. [pwMS 46] |
| Experiences with Nutrition and Supplements\Starting new habits\Daily impacts\Positive attitude towards food | I used to live on instant noodles completely. For a while, I lived on nothing but cornflakes. Or junk food in general, frozen pizza and so on. Now, to be honest, I don't feel like dumping that stuff into my body anymore either, but I celebrate food a little bit more. [...] The MS was definitely the trigger, which is why I started doing it. And now I wouldn't go back either. [pwMS 33] |
| Experiences with Nutrition and Supplements\Starting new habits\Daily impacts\Seeing MS as an opportunity | Well, the diagnosis helped me to start a sensible diet. [pwMS 20] |
|  | MS was definitely the trigger that made me start doing it. [pwMS 33] |
| Experiences with Nutrition and Supplements\Starting new habits\Daily impacts\General health benefits | I also lost thirty kilos now, because of the diet and because of the sports that I then began as well. [pwMS 06] |
|  | Now I feel a lot better than before.”. [pwMS 45] |
| Experiences with Nutrition and Supplements\Starting new habits\Daily impacts\MS-specific health benefits | Yes, so I have a relatively healthy lifestyle change. So, I don't drink alcohol, I don't smoke. I think that makes a lot of difference and I've been eating a vegan diet for about 6-7 years. And I also notice that I think that helps a lot, because these animal fats are simply not good for this inflammation at all. I think I do a lot because my relapse frequency is actually always 2, 3 years. So now I don't always have [a relapse] every two months. [pwMS 35] |
|  | I can't judge whether this has a positive or negative influence on MS, because I don't know what it would look like if I didn't do it. The only thing I know is that during the time when it was left the old way, MS developed [...] and evolved. [pwMS 09] |
| Experiences with Nutrition and Supplements\Starting new habits\Daily impacts\Barriers leading to easing/termination\Diet too strict | In the beginning [...] I tried to change my diet completely. But I think that was too radical and that's why it didn't work. And then I just went back to old habits a bit. [pwMS 45] |
|  | But at some point [...] the inner strength was no longer big enough to keep it up, when you feel like eating a steak or meat now and then, or a dessert for a change.” [pwMS 29] |
| Experiences with Nutrition and Supplements\Starting new habits\Daily impacts\Barriers leading to easing/termination\Uncertainty of results | I never had anyone or anything to support my theories, which were only experiences. There was nothing that would have told me: Yes, nutrition and MS, there is a connection.” [pwMS 36] |
| Experiences with Nutrition and Supplements\Starting new habits\Daily impacts\Barriers leading to easing/termination\Negative emotions | At the beginning I completely gave up meat for quite a while. But I noticed that it was not good for my mood [...] I tried it. But I become unhappy with it. Even if I do it for a longer time, I am unhappy. [pwMS 33] |
|  | And I also, especially in the beginning, because I really wanted to do everything to somehow positively influence [MS], I also no longer snacked, so really not at all, tried to avoid sugar as much as possible. I really didn't eat any sweets for a whole month, and at first, I always had a guilty conscience when I actually ate a piece of chocolate or something like that. [...] At some point, Easter came, yes, and everyone else was of course feasting and you sat there like that and you're not really allowed to eat that now. And when I ate it, it was immediately like, what does this do to me now, does this trigger something now? [pwMS 06] |
| Experiences with Nutrition and Supplements\Starting new habits\Daily impacts\Barriers leading to easing/termination\Unfulfilled expectations | At some point I had my control MRI appointment and had a new inflammation. [...] So somehow it didn't really help. And that was the trigger for me to go about it in a more relaxed manner. [pwMS 06] |
| Experiences with Nutrition and Supplements\Maintaining previous habits\Influencing factors\Desire for self-determination | I think the illness already determines so much of your life. I’m not going to be told what I should eat or what I shouldn't eat, or when I should do what kind of sports. I still decide that myself. [pwMS 10] |
| Experiences with Nutrition and Supplements\Maintaining previous habits\Influencing factors\Desire for daily life without restrictions\Desire for daily life without restrictions | It limits you. Whenever there are parties or something like that, you are always the one who has to say no and who is always left out somehow. You somehow exclude yourself a little bit, too. [pwMS 44] |
| Experiences with Nutrition and Supplements\Maintaining previous habits\Influencing factors\Missing guidelines for the “right” nutrition | There's just a little lack of a good approach that you could use as a person with MS, and to say, here's your guideline. [...] I also think it's difficult to somehow make a guideline for everyone. Because there is not going to be a one-size-fits-all guideline. [pwMS 45] |
| Experiences with Nutrition and Supplements\Maintaining previous habits\Daily impacts\Satisfaction | It may not be something that all people understand, why my visit to McDonald's is associated with quality of life, but for me that’s the case [pwMS 13] |
| Experiences with Nutrition and Supplements\Practiced lifestyle habits\Avoidance/Reduction of certain food\Animal products | I've been a vegan for three years now. So not quite a strict vegan. If there's an egg in the cake, I eat a piece of cake. That's not the problem. Well, eating eggs just like this now, that's not possible. I used to enjoy eating oven cheese. I can't do that anymore. [pwMS 37] |
|  | Yes, in 2007, when I was diagnosed with MS, I was also interested in what could be done to prevent further episodes of inflammation. It was then clear that the diet should be changed, perhaps to more vegetarian. And that's what I did. So I eat, I'll say 98% vegetarian. What I don't eat at all is pork. [...] What I also eat is fish. [pwMS 25] |
|  | I have looked at what you should eat and what not. According to the Internet, MS patients should not eat dairy products with little fat. So I just switched to other milk, for example. [pwMS 41] |
| Experiences with Nutrition and Supplements\Practiced lifestyle habits\Avoidance/Reduction of certain food\Carbohydrates | So I also tried the ketogenic diet [...] Well, that's a very low-carbohydrate diet, which means under 30 grams of carbohydrates. You really have to count every vegetable. [pwMS 32] |
|  | You should avoid carbohydrates, because carbohydrates would contribute to it, could - this is not proven, but is suspected - okay, that it goes better without carbohydrates. So I left out the carbohydrates. I don't think that's a big deal. Then you leave out the potatoes, the pasta and the rice and just eat the roast pork without. That's okay. Well, and the vegetables and so on, the fruit, you still have to eat them. [pwMS 09] |
| Experiences with Nutrition and Supplements\Practiced lifestyle habits\Avoidance/Reduction of certain food\Alcohol | There just came a point where I no longer saw the added value in alcohol. Because the next day I feel even shittier than I did before. And that can start very quickly, so even if I've only drunk two small bottles of beer or so, I'm completely shit the next day. And I think it's more because of the medication I take. And that's why it's a bit more the added value that I no longer see in alcohol and the benefit. So it has no benefit, but to say, if I feel like shit the next day, then it's not worth it at all. [pwMS 45] |
| Experiences with Nutrition and Supplements\Practiced lifestyle habits\Avoidance/Reduction of certain food\Linoleic acids | I have changed my diet. [...]I eat a diet low in linoleic acid, because it is precisely this linoleic acid that can be held responsible for destroying the myelin layer. That is not certain, but that means I leave out legumes, I leave out pork, I leave out egg yolks and also dairy products predominantly. [pwMS 50] |
| Experiences with Nutrition and Supplements\Practiced lifestyle habits\Specific diet programs\Mediterreanean diet | Then I think that's great with Mediterranean cuisine, which you can then do, so I also found that very nice. So Mediterranean cuisine simply means more plant matter, less meat. Meat has never been a problem for me anyway. [pwMS 28] |
| Experiences with Nutrition and Supplements\Practiced lifestyle habits\Specific diet programs\Paleo | Terry Wahls means first of all the renunciation of lactose, all dairy products actually, and secondly, the renunciation of all gluten-containing products, that is, everything that is grain. If you ever walk through a supermarket, you'll notice that a relatively large amount of it has to do with either grains or lactose. Accordingly, it's quite a big step and [...] there are three levels. There's a basic program, a Paleo program, it's called, and then there's Paleo-plus. It's based on this Paleo diet, but I don't think it's strictly speaking a Paleo diet, but it's definitely inspired by it. In this basic variant, you just do without the two things mentioned, eat nine cups [...] fruits and vegetables every day and also meat [...] and if cereals, then pseudo cereals, so gluten-free products, nuts and something like that are of course also somehow part of it. [pwMS 36] |
| Experiences with Nutrition and Supplements\Practiced lifestyle habits\Specific diet programs\Intermittent fasting | What I have done before and will do again now is interval fasting, because that is also being tested in this nutrition study at Charité. [pwMS 32] |
| Experiences with Nutrition and Supplements\Practiced lifestyle habits\Specific diet programs\Individual healthy diet | Healthier diet, paid more attention to it, lots of vitamins, lots of fiber, just tried to eat healthy and balanced. Little fat. [pwMS 08] |
|  | I'm not saying I don't eat pork at all anymore. But I know that pork is the unhealthiest of all. Of course, even cheap poultry is no longer eaten. But I also don't say now, if I'm just at the barbecue, and then a piece of meat is over at the grill, and you ask, do you want to eat that, then I say yes, why not. So depending on how the mood is, of course. [...] I avoid milk a bit more. But even that only scaled back. I've switched more to soy products. So basically I started to eat healthier. [...] MS was definitely the trigger, which is why I started. [pwMS 33] |
| Experiences with Nutrition and Supplements\Practiced lifestyle habits\Vitamins and Supplements\Vitamin B, D | So I started with vitamin D. [...] At first I probably kind of took a supplement with 20000 units every three weeks, so I had 1000 units daily. Then I started taking that weekly. And since there are now also ideas with these high-dose therapies, [...] and in my circle of acquaintances people use this and we have also talked a lot about it in the context of the self-help group, I actually take 5000 units daily. [pwMS 32] |
|  | I also take additional medications from the doctor's side, vitamins, vitamin B and vitamin D preparations, which are supposed to counteract the fatigue somewhat, which are supposed to make you a bit fitter, which simply show a bit of effect. Whether they show it or not, I don't know. [pwMS 25] |
| Experiences with Nutrition and Supplements\Practiced lifestyle habits\Vitamins and Supplements\Magnesium | Magnesium, [...] I take it for support. [pwMS 04] |
| Experiences with Nutrition and Supplements\Practiced lifestyle habits\Integration of certain food\Spinach | For a while I also often took fresh baby spinach leaves. Simply doused with hot water and then mixed with tomato paste and pepper and then eaten with olive oil. […] So a week of that was like a week in the hospital. [pwMS 18] |
| Experiences with Nutrition and Supplements\Practiced lifestyle habits\Integration of certain food\Ginger | I have tried anti-inflammatory diet for a while, or I still do. That was for me in the form of ginger and just take a bite of it like an apple. [pwMS 18] |
| Experiences with Nutrition and Supplements\Practiced lifestyle habits\Integration of certain food\Cinnamon | [I] eat my muesli [...] then with cinnamon, especially Ceylon cinnamon, because it's anti-inflammatory. [pwMS 50] |
| Experiences with Nutrition and Supplements\Practiced lifestyle habits\Integration of certain food\Curcuma | But there were just things, I tried every now and then [like] eating fresh curcuma root. Yes, I wasn't sure to what extent the strongly colouring stuff would actually change the colour of the teeth. And I didn't really feel like doing that. Then I just cut thin slices off, put it in between small cocktail tomato. And then with pepper it went down easier. [pwMS 18] |
| Experiences with Nutrition and Supplements\Practiced lifestyle habits\Integration of certain food\Green tea | Green tea in the morning, noon, night. I've always drunk green tea, but not to that extent. It makes sense to me to the extent that it's just part of my daily routine to drink green tea instead of coffee. And the other point is that the effects of the antioxidants in tea, I think, can also be seen quite positively in MS. [pwMS 26] |

| Experiences with Exercise and Physical Activity\Starting new habits\Influencing factors\Information and advice | [I] also took part in a sports study a few years ago. And since then, [...] I try to exercise as regularly as possible. [pwMS 19] |
| --- | --- |
| Experiences with Exercise and Physical Activity\Starting new habits\Influencing factors\Active disease management | But then came the day when I thought to myself, that makes sense somehow. If you once more have to go to the hospital […] while being weak, it would indeed be nice if you somehow had a fitter basis, so that you don't completely collapse and have to start again from scratch. Well, then the jogging started.” [pwMS 06] |
|  | Because a lot of people said, "Yes, in a few years you'll end up in a wheelchair," and of course I didn't want that. For God's sake. And then I just did everything I could to make it better somehow. [pwMS 14] |
|  | The MOTOmed is important for me to just keep moving the legs through [...] so the joints don't stiffen up. [pwMS 29] |
|  | Yeah, I started doing yoga to stretch a lot to keep the tendons flexible and also that the muscles just stay flexible. [pwMS 04] |
|  | You should practice falling so that in the situation when it happens, you can roll off a bit better. And it is very important to be able to get up again. [pwMS 03] |
| Experiences with Exercise and Physical Activity\Starting new habits\Influencing factors\Coping with stress | So, effectively for stress management, well, that's where I actually have sports. [pwMS 50] |
| Experiences with Exercise and Physical Activity\Starting new habits\Influencing factors\Desire for social support | In our neighbourhood in Ulm, there is a sports club that also has a sports department for the disabled, which offers wheelchair sports, among other things. [...] there is also a group of severely affected people who do more stretching exercises, coordination exercises and, very importantly, this subsequent social gathering after the sport. I think it's very important to actually maintain social contacts. [pwMS 29] |
|  | What’s really great for people with MS is Aikido [...] You are more or less amongst like-minded people and can exchange ideas. [pwMS 01] |
| Experiences with Exercise and Physical Activity\Starting new habits\Daily impacts\Feeling of strength, perseverance, relief | Then you have also developed a certain discipline and a certain strength, when you want to achieve this and that. And that helps with such an illness. [pwMS 37] |
|  | [When riding, I] also really forgot about work. I didn't take anything home with me then. [...] At that moment, the illness was very far away. [pwMS 32] |
| Experiences with Exercise and Physical Activity\Starting new habits\Daily impacts\Impact on MS-specific health\MS-specific health benefits | Well, I always had the impression that [climbing] is really good for me, because it simply has a lot to do with coordination and with concentration, especially. [...] less with strength [...] It's actually more about movement intelligence and yes, that's actually ideal for MS. [pwMS 36] |
|  | I went back to the gym and then I really did strength training [...] then I trained on good equipment, also really with really heavy weights, and there I noticed, over a good quarter of a year, that walking was better again. [pwMS 22] |
|  | But I can now manage to get up again on my own, that is to say, to lift myself up, and then to stand on my legs, then straighten my upper body [pwMS 03] |
|  | Sport helps me a lot with MS. I have good musculature, so I can still move relatively well for my circumstances. But of course, I also have a lot of mass for cramps because of all the muscles. Spasticity. But that's just the way it is. You can't get one without the other."[pwMS 37] |
| Experiences with Exercise and Physical Activity\Starting new habits\Daily impacts\Perceived adverse effects leading to easing/termination | But when I noticed that the symptoms of the recent relapses became stronger under strain, that really distressed me, that scared me, and I then felt less and less well. And then, when you start crying on the treadmill because your legs feel wrong, because your arm feels funny, then that's not it. [pwMS 49] |
|  | The problem is that when I did sports, I always felt worse. So, unfortunately, sport was not an option for me. [pwMS 47] |
|  | I don't think I can cope with sports at the moment, [...] I feel like I'm annoying the MS with it. [...] It hits the optic nerve or the vision, [...] since I've been doing these sports measures again. [pwMS 40] |
| Experiences with Exercise and Physical Activity\Maintaining previous habits\Influencing factors\Not being active before | I was the little fat one at school and sports was no fun all of my life. Taking up sports only after the diagnosis is a double challenge, simply because exercise [...] has never been much fun for me and now [exercise] is even limited. That means it’s a rough ride. And I haven't made the journey - yet. [pwMS 13] |
| Experiences with Exercise and Physical Activity\Maintaining previous habits\Influencing factors\Possibility to modify previous exercises | For instance, for me it would be like this, because of the dizziness, I can use a bicycle ergometer instead of riding a bicycle, [...] if I turn around, I'll probably end up in the next ditch, but today these are such minor things for me where I say: Well, then it doesn't work anymore. But you can replace it with small movements, and you have to be a bit creative." [pwMS 05] |
|  | You don't do a ten-kilometer tour a day, but only three kilometers, with breaks accordingly. And you take the cable car up instead of walking up or down. [pwMS 30] |
| Experiences with Exercise and Physical Activity\Maintaining previous habits\Influencing factors\Desire for fun | And I also believe, and this is the most important thing, that you should just do what you feel you’re up to [...]. And just because everyone else is doing yoga, and they also say that it helps you so much, that means that you yourself are the one who wants to do it. And if you just want to lie on the couch at that moment, then that’s what you should do. [pwMS 34] |
| Experiences with Exercise and Physical Activity\Practiced lifestyle habits\Endurance sports\Biking | I live in the middle of town now, which means I try to do a lot by bike. But I also bought an e-bike two years ago. I work [...] ten kilometers away. It's normally no problem by bike if it would not go steadily uphill. [...] That's why I bought an e-bike and when it's nice weather, I ride my bike. [pwMS 31] |
| Experiences with Exercise and Physical Activity\Practiced lifestyle habits\Endurance sports\Walking, Jogging, Hiking | I go for a lot of walks. That's what I like to do. In fact. Two hours at a time, for example, walking around a bit. [pwMS 43] |
|  | We are both mountain people, me too, and in the beginning, we were still running and hiking a lot. And were much in Austria, in the mountains. Well, we still did that, but now it was just the Black Forest. But then I just walk and take breaks accordingly. [...] then you know, okay, you don't do a ten-kilometer tour a day, but only three kilometers, with breaks. And you take the cable car up instead of walking up or down. [pwMS 30] |
|  | With MS, I just really started jogging. [pwMS 18] |
| Experiences with Exercise and Physical Activity\Practiced lifestyle habits\Endurance sports\Dancing | I do something like Zumba, [...] you get body awareness. And that body awareness is important to me. [pwMS27] |
| Experiences with Exercise and Physical Activity\Practiced lifestyle habits\Endurance sports\Fitness | in the winter i go to the gym twice a week and do full-body workouts every day and if i still have time and feel like it, i get on the bike ergometer in the gym and ride the bike for another hour. [pwMS 22] |
| Experiences with Exercise and Physical Activity\Practiced lifestyle habits\Resistance sports\Climbing | [I have] started climbing with my husband. [...] Since I think it's a totally great sport, really incredible for MS as well. Well, I always had the impression that [climbing] is really good for me, because it simply has a lot to do with coordination and with concentration, especially. [...] less with strength [...]. It's actually more about movement intelligence and yes, that's actually ideal for MS. [pwMS 36] |
| Experiences with Exercise and Physical Activity\Practiced lifestyle habits\Resistance sports\Fitness | I went back to the gym and then I really did strength training [...] then I trained on good equipment, also really with really heavy weights, and there I noticed, over a good quarter of a year, that walking was better again. [pwMS 22] |
| Experiences with Exercise and Physical Activity\Practiced lifestyle habits\Resistance sports\Horse riding | I do horseback riding and have always done that a lot, actually every day. [...] Horseriding is wonderful. Apart from the fact that it relaxes me, that it's basically my yoga, you're just out in the fresh air and when you are horseback riding, you use all the muscle groups. [pwMS 06] |
| Experiences with Exercise and Physical Activity\Practiced lifestyle habits\Water sports\Aquajogging | This is also a group of 20 ladies where I am, I am the youngest, but yes, the only one who has a handicap and that is really 45 minutes full power in the water, as far as you can go and that helps immensely. I feel really good afterwards, because that's ideal for running, I mean running in the water, so that I can move my legs properly. [pwMS 07] |
| Experiences with Exercise and Physical Activity\Practiced lifestyle habits\Water sports\Swimming | I can swim very very well. And well, I can still do that, and it's a lot of fun. [...] you are in the water, maybe until you get into the water it is difficult. [...] But when I'm in the water, then I'm free. Then it's great. Then it's awesome. And then I also like swimming very much. I can crawl well. And then I swim forever. [pwMS 11] |
| Experiences with Exercise and Physical Activity\Practiced lifestyle habits\Water sports\Standup Paddling | I've now started doing a bit more sport again somehow. [...] I bought my wife a stand-up paddle, a board (laughs). But also with the background, because I know from the clinic again that it is quite good for the sense of balance, for the deep muscles. Even if I can't stand on it now, maybe not yet, but I can kneel on it and paddle. And kneeling and paddling is also a challenge. [pwMS 11] |
| Experiences with Exercise and Physical Activity\Practiced lifestyle habits\Water sports\Water gymnastics | So water gymnastics was great. I was always the youngest among the participants, but it really helped. Because you feel weightless, you don't have those balance problems, so I can really recommend that. [pwMS 47] |
| Experiences with Exercise and Physical Activity\Practiced lifestyle habits\Movement exercises and meditation\Aikido | What is really great is Aikido for people with MS. Unfortunately, we have too few participants, for whatever reason, but in any case, it's great. It trains your balance; you are more or less among like-minded people and you can exchange ideas. So it's a great thing. [pwMS 01] |
| Experiences with Exercise and Physical Activity\Practiced lifestyle habits\Movement exercises and meditation\Qigong | Qigong is still the first choice, but as I said, the problem was also the offer, because you do not want to spend too much money. [...] It was also so that she also responded to me, the teacher, is that yes. If I say, no, I can't do that standing up, then she said, "Then do it like that while sitting down." That was actually very good. And in the end, it's such quiet movement sequences, where you partly have stretching. [pwMS 31] |
| Experiences with Exercise and Physical Activity\Practiced lifestyle habits\Movement exercises and meditation\Yoga | And now I have found another new sport. And that is a yoga in a sitting position. That's also just for people with MS, which is great for me. So in squat. And I do that now on Tuesday evenings. [pwMS 19] |
| Experiences with Exercise and Physical Activity\Practiced lifestyle habits\Movement exercises and meditation\Pilates | So, Pilates is a kind of gymnastics. I think in a different context... Yes, actually one would say 'floor gymnastics' or standing, but it is actually a form of gymnastics with some yoga elements in it. Now not these spiritual relaxation things from yoga, but just the physical exercises. [...] It's always about the powerhouse, the torso muscles, [...] exactly my weak point and therefore it fits great and it's not with any warm-up where you have to jump. I can't do that. [pwMS 20] |
| Experiences with Exercise and Physical Activity\Practiced lifestyle habits\Sports with specific equipment\Wheelchair sports | What I continue to do every week actually is a participation in wheelchair sports. In our neighbourhood in Ulm, there is a sports club that also has a sports department for the disabled, which offers wheelchair sports, among other things. Once more performance-oriented with wheelchair basketball, which I can no longer do anyway with my strength and motor skills. But there is also a group of severely affected people who do more stretching exercises, coordination exercises and, very importantly, this subsequent social gathering after the sport. I think that's very important for maintaining social contacts. [pwMS 29] |
| Experiences with Exercise and Physical Activity\Practiced lifestyle habits\Sports with specific equipment\Paragliding | There are many things I could not do, but then I found new hobbies, skydiving, at the moment only piggyback, everything else I'm not allowed yet, but I'll work towards that again. [pwMS 05] |
| Experiences with Exercise and Physical Activity\Practiced lifestyle habits\Sports with specific equipment\Sports with MOTOmed | The MOTOmed is important for me to just keep moving the legs through. I can no longer actively pedal, but it is at least as important that the legs are moved at all, so the joints don’t stiffen up. I can no longer lift my legs into and out of the MOTOmed myself. I need support for that. But in spite of everything it is important that it is done every week and regularly if possible. [pwMS 29] |
| Experiences with Exercise and Physical Activity\Practiced lifestyle habits\Sports with specific equipment\Sports with Powerplate | I've been doing Powerplate Training for a year and a half now. Powerplate training is actually a vibrating plate on which you can do an incredible amount of exercises. At first I totally smiled at this thing, until at some point I had such sore muscles that I thought: What, there are muscles there too? That was such a new thing that I actually got to know. [...] And because of this constant rotation or vibration, it appeals to the whole body if you have the right posture. I think that's mega-great. [pwMS 05] |
| Experiences with Exercise and Physical Activity\Practiced lifestyle habits\Fall prevention | Now last year I started taking a course in fall prevention. I walk with a cane or with a walker, but falling is an important topic, or let's say preventing that. [...] And yes, we start with warm-up exercises. It's sixty minutes and it's insanely intense. I have puppy status there. I always pull up a chair and sometimes I don't even stand during the warm-up. [...] So you have to practice falling, you should practice falling, so that in the situation where it happens then you can roll a little better. And it's incredibly important to be able to get up again. [...] When I started this fall prevention course, that was four or five months ago, I couldn't get up from the floor. I couldn't manage it. [...] But now I'm able to get up again on my own, I can get myself up so far to then use my own legs to get my upper body up. [pwMS 03] |
| Experiences with Stress Management\Starting new habits\Influencing factors\Information and advice | With relaxation I have changed a lot, just the last four years. I have started meditating with all the knowledge behind it, why it is so helpful. There are many research results available on this topic. [pwMS 02] |
|  | [In 2011,] my sister, who is attached to Buddhism, said: "Read this book.". And that was the book by Jon Kabat-Zinn, founder, inventor, whatever you want to call it, of the mindfulness-based stress reduction program. And that's when I read this and then I also dealt with it more intensively. [pwMS 32] |
| Experiences with Stress Management\Starting new habits\Influencing factors\Active disease management | High stress level [...] immediately has a physical effect on me. [...] I then stumble again, my dizziness immediately appears, my hands are even more numb. [...] Everything that has been there before [...] flares up. [...] For me, that is always immediately the sign [...] to somehow put boundaries around me, hand things over and try to get periods of rest for myself. [pwMS 46] |
|  | Not only I was scared after this diagnosis, of course, but my wife was too. Life changes. Questions arise. My questions were: "What can I still achieve?" [...] For my wife, it was certainly the case that for her the question was: "For God's sake, what do I have to start doing now? Is he going to be a nursing case like this within six months?" That means, not only did my life completely change, but my wife's life completely changed as well. […] When we realized that my wife [...] was also confronted with fears due to the diagnosis, we again turned to the German Multiple Sclerosis Society and were given the opportunity to undergo couples therapy. [pwMS 13] |
| Experiences with Stress Management\Starting new habits\Influencing factors\Desire for mental health benefits | There were already very clear signs that I was not well. I was, I think, very, very sad for years. Extremely sad [...] At that time I had [a] phase [in which I] had panic attacks again and again [...] I think also because I had the feeling that I had no one to talk to, I had the feeling that maybe such a therapy would not be a bad idea. [pwMS 36] |
|  | And then I often took weekends, relaxation weekends, to do something in that direction. And I tried to bring my life into balance a bit. So not to let many things [...] get to me like that. Not to let myself get stressed. So to try to reduce my stress. [pwMS 08] |
| Experiences with Stress Management\Starting new habits\Influencing factors\Desire for social support | f you don't say that right out and also give the reason behind it, then you put yourself in a very strange role as a victim, because then the pity comes. [pwMS 26] |
|  | It has to be said that the situation is such that I have multiple sclerosis, only then my counterpart can respond to that without thinking: "He must be lazy."[pwMS 13] |
| Experiences with Stress Management\Starting new habits\Daily impacts\Mental health benefits\ | So I've gotten into the habit of listening to audiobooks, funnily enough, so that totally relaxes me. [pwMS 10] |
|  | When I notice that I'm running short of time for something, I call and say “I don't know if I'll make it on time.” And then there's usually enough time, but there’s no pressure on me anymore. [pwMS 42] |
| Experiences with Stress Management\Starting new habits\Daily impacts\development of coping strategies | And that's the way to deal with it, to arrive within this illness, to say: Okay, I'm not looking for the possibility of not having the illness, but I have it now and it won't go away, it will be there all my life, it's part of me. And I think psychotherapy is very important for dealing with that. Well, it sure helped me. [pwMS 22] |
|  | In just dealing with it when symptoms appear [...], well, meditation has helped me a lot [...] not to panic directly, to perceive it first, to feel one’s body somehow or to try to feel it. [pwMS 36] |
|  | And what's also quite super is to take one’s time and say: "Also be grateful that we're doing, well, good right now, and that we don't really need anything. [pwMS 15] |
|  | You exchange ideas about what is good, because the others have other deficits or you know more, because you've been around longer [pwMS 28] |
|  | And I can also see something positive in MS. I think it was also a cry for help from my body, to take care of myself. And yes, to also work on myself. To take care of myself. [pwMS 34] |
| Experiences with Stress Management\Starting new habits\Daily impacts\Social support and interaction | Other people [have] also said to me: You have actually become a better person. I also became more sensitive myself. Today, I can also listen better. [pwMS 22] |
| Experiences with Stress Management\Starting new habits\Daily impacts\Barriers leading to easing/termination\Unfulfilled expectations | Yes, I just went there: ‘Well, if you can't make my illness go away, then you can't help me either'. [pwMS 02] |
| Experiences with Stress Management\Starting new habits\Daily impacts\Barriers leading to easing/termination\unfortunate experience | The people were much older than me and, unfortunately, some of them were already very limited. That actually scared me at that moment rather than helped me. [...] And it rather frightens you [pwMS 43] |
|  | Then I was in a support group on Facebook. [...] So it really just dragged me down, because everyone was just complaining about their problems and I thought, that's not a support group, that's a "we're-pulling-ourselves-down-together". [pwMS 50] |
| Experiences with Stress Management\Starting new habits\Daily impacts\Barriers leading to easing/termination\spasticity | You're supposed to strain the muscle and then let it go again, that doesn't work at all, then for me it just closes up. So spasticity is a symptom that is very sensitive to stress. So the more stress you have, the more you exert yourself, the stronger the spasticity becomes, and that's when you actually need relaxation, and for me, this was just tension. [pwMS 28] |
| Experiences with Stress Management\Practiced lifestyle habits\Relaxation methods\Feldenkrais | I do Feldenkrais [...]. Feldenkrais is something, no matter how severe that you are disabled, anyone can do Feldenkrais. Feldenkrais is a method to achieve a lot with very small exercises. I've been doing it since I have MS, since 2007, and I think it's the best thing existing. [...] So in Feldenkrais there is a therapist who achieves an exercise module, which you then do very slowly. You always start to repeat the exercise slowly and by doing it on one half of your body first and then trying to scan your body again, to see how it has changed, and then the second part, the other half of your body, you suddenly notice that it becomes loose. [...] So that's phenomenal. There are great exercises. [...] It's just pure relaxation. [pwMS 28] |
| Experiences with Stress Management\ Practiced lifestyle habits\Relaxation methods\PMR | Muscle relaxation according to Jacobsen [...] has also helped me well when I was very exhausted. [...] In Jacobsen muscle relaxation, you tense different muscle groups in a fixed sequence. [...] And then you let go. And this letting go, you consciously relax and try to relax a little more and more [...]. And then also to feel how it feels. [pwMS 32] |
| Experiences with Stress Management\ Practiced lifestyle habits\Relaxation methods\MBSR | Many years ago I took an MBSR course [...] This is an abbreviation for Mindfulness Based Stress Reduction [...] developed by Jon Kabat-Zinn. [...] I think it was originally developed for people with chronic diseases, incurable diseases, who have to learn to live with symptoms permanently and to endure symptoms. [...] [The] Bodyscan [is] a guided journey through the body, you could say. It's actually about feeling the individual parts of the body bit by bit and making contact with the individual parts. As we progressed, we also began to meditate. [...] I really did it extremely regularly for one or two years. So I really sat down and meditated every day [pwMS 36]. |
| Experiences with Stress Management\ Practiced lifestyle habits\Relaxation methods\Meditation | With relaxation I have changed a lot, just the last four years. I have started meditating with all the knowledge behind it, why it is so helpful. [pwMS 02] |
| Experiences with Stress Management\ Practiced lifestyle habits\Coping methods | [We] got the opportunity to do couples therapy there. [...] That's nothing more than having an opportunity to talk. [pwMS 13] |
|  | There were already very clear signs that I was not well. I was, I think, very, very sad for years. Extremely sad [...] At that time I had [a] phase [in which I] had panic attacks again and again [...] I think also because I had the feeling that I had no one to talk to, I had the feeling that maybe such a therapy would not be a bad idea. [pwMS 36] |
| Experiences with Stress Management\ Practiced lifestyle habits\Living with pets | We have two dogs at home. [...] The animals are a very great benefit for me. They give me a lot of strength and a lot of energy and are very often there for me more than perhaps other people [...]. And the animals have a great understanding for me and for my illness, because they obviously sense when I'm not feeling well. And then they also tend to lie down. So they are. Also, rather relaxed when they feel that I am not really well and that I am very tired. That's a very pleasant behaviour and it's also very good for me. [pwMS 25] |
| Experiences with Stress Management\ Practiced lifestyle habits\Daily routines\Reading | Well, to cope with stress, I usually do crossword puzzles and Sudoku for myself. And of course I read the newspaper every morning. [...] That's also part of the relaxation for me, to read the newspaper in peace, so that I can leave the house around 8:00 a.m. [pwMS 29] |
| Experiences with Stress Management\ Practiced lifestyle habits\Daily routines\Gardening | On weekends, we have a weekend house outside Munich and a garden; and the garden has become strongly therapeutic for me. Mowing the lawn, surprisingly, has become a very meditative activity. The reason is that a lawn mower, you hold on to it and when you hold on to it and walk back and forth behind the lawn mower, then walking is not so difficult and that ensures that my head switches off and that I just don't think about MS. [pwMS 13] |
| Experiences with Stress Management\ Practiced lifestyle habits\self-care methods\Support groups | Then I was in a support group on Facebook. [...] So it really just dragged me down, because everyone was just complaining about their problems and I thought, that's not a support group, that's a "we're-pulling-ourselves-down-together". [pwMS 50] |
|  | I'm in a support group. Exactly. And that is also quite helpful. It's a national one. And, yes, there [...] we exchange ideas quite intensively. [pwMS 32] |
| Experiences with Stress Management\ Practiced lifestyle habits\self-care methods\Recognizing limits | If I want to do more now, then I notice my numb spots again very intensively, then I know: "Aha, that's the alarm again." Then I try to sit down somewhere nice again when I'm not strong. Yes, so a cup of coffee in peace and just sit down and it's all again, yes, it's already not so bad. Then I say, okay, yes, now we have taken care of us together again. [pwMS 15] |
|  | I found it very, very difficult to take a step back and just slow down and look after myself. I think it took me a year and a half before I was able to say, "Okay, you're not going back to work, you don't need to worry about anything now, you don't need to be under pressure or anything, but just take it easy now" [pwMS 48]. |
| Experiences with Stress Management\ Practiced lifestyle habits\self-care methods\Optimism and openness | And when I look back over the years, I realize that I have such a basic trust, I obviously do. This way of thinking, there's always a reason for something. If one thing doesn't work, something else will. Such a confidence, yes, a confidence. Such a basic confidence. That's definitely very helpful. [pwMS 42] |
|  | I no longer hike now. Now I can go back and forth with the wheelchair. But it's basically always something else that you then gain again. And that's actually what I find so beautiful. You always lose something, but you also gain a lot. And especially on the interpersonal level, you gain an incredible amount. [pwMS 23] |
|  | What I find important is that you play with open cards, that you are not ashamed of this disease, because no one can do anything about it. It is also not a bad thing, even if the disease is not as friendly to you as it is to me at the moment. Nevertheless, play with open cards and approach the disease in this way, because only then can you tackle it and do something about it. [pwMS 50] |
