# Supplementary material for: Experiences of persons with Multiple Sclerosis with lifestyle adjustment–A qualitative interview study
Source: PLoS One. 2022 May 27;17(5):e0268988. doi: 10.1371/journal.pone.0268988 (PMC9140290; doi:10.1371/journal.pone.0268988)
Supplement: Attachment — Submitted filename: Response to Reviewers 2.docx [file pone.0268988.s005.docx]

**Subject: Edits requested on the submission PONE-D-21-10862R1**

Dear Ms. Rose Ann Joyce Sagun Puetes,

Thank you for giving us the opportunity to submit a revised draft of our manuscript entitled “Experiences of Persons with Multiple Sclerosis with Lifestyle Adjustment - A Qualitative Interview Study” to PLOS ONE.

We believe that the paper has significantly improved with your help. Please find below a point-by-point response and let us know if you have any further concerns or questions.

Best wishes

Saskia Elkhalii-Wilhelm

**Point by point response**

**Editor’s comments:**

**Comment 1**: Please consider the items from either COREQ (Tong et al) or SRQR (o'brien et al) and in particular the method section and any missing items of information e.g., strategies to enhance quality. If I have missed this apologies.

**Response 1:** We appreciate this advice and agree about the importance of referring to specific criteria for reporting qualitative data to enhance quality. Now we have clarified how we specifically referred to the COREQ checklist from Tong et al. (2007) in a newly created “S2 Appendix”.

**Comment 2:** Could you update the Braun and Clarke reference noting they have a website and recent articles and the name of the analysis has changed.

**Response 2:** We thank you for taking the time to evaluate our manuscript and for this valuable comment. Indeed, Braun and Clarke call their approach also ‘reflexive thematic analysis’ to show that this approach is quite theoretically flexible to address different types of research questions. Thematic analysis can be approached in an inductive, deductive, semantic, latent realist or in a constructionist way (Braun and Clarke, 2022). The coding and theme development in our article was directed by existing concepts, on the one hand, and by the content of the data, on the other hand. We added that this form of analysis is reflexive and updated the reference list by adding recent articles of Braun and Clarke (2019, 2021) and referring to their website www.thematicanalysis.net on p. 7, lines 157-158:

“Data were analyzed inductively and deductively according to the six-step (reflexive) thematic analysis of Braun and Clarke [43-46]”
